# From whole bodies to single cells: A guide to transcriptomic approaches for ecology and evolutionary biology

**DOI:** 10.1111/mec.17382

**Published:** 2024-06-10

**Authors:** Katja M. Hoedjes, Sonja Grath, Nico Posnien, Michael G. Ritchie, Christian Schlötterer, Jessica K. Abbott, Isabel Almudi, Marta Coronado‐Zamora, Esra Durmaz Mitchell, Thomas Flatt, Claudia Fricke, Amanda Glaser‐Schmitt, Josefa González, Luke Holman, Maaria Kankare, Benedict Lenhart, Dorcas J. Orengo, Rhonda R. Snook, Vera M. Yılmaz, Leeban Yusuf

**Affiliations:** ^1^ Amsterdam Institute for Life and Environment Vrije Universiteit Amsterdam Amsterdam The Netherlands; ^2^ Division of Evolutionary Biology LMU Munich Planegg‐Martinsried Germany; ^3^ Department of Developmental Biology, Göttingen Center for Molecular Biosciences (GZMB) University of Göttingen Göttingen Germany; ^4^ Centre for Biological Diversity University of St Andrews St Andrews UK; ^5^ Institut für Populationsgenetik Vetmeduni Vienna Vienna Austria; ^6^ Biology Department Lund University Lund Sweden; ^7^ Departament de Genètica, Microbiologia i Estadística Universitat de Barcelona Barcelona Spain; ^8^ Institut de Recerca de la Biodiversitat (IRBio) Universitat de Barcelona Barcelona Spain; ^9^ Institute of Evolutionary Biology CSIC, UPF Barcelona Spain; ^10^ Department of Biology University of Fribourg Fribourg Switzerland; ^11^ Functional Genomics and Metabolism Research Unit, Department of Biochemistry and Molecular Biology University of Southern Denmark Odense Denmark; ^12^ Institute for Zoology/Animal Ecology Martin‐Luther‐University Halle‐Wittenberg Halle (Saale) Germany; ^13^ School of Applied Sciences Edinburgh Napier University Edinburgh UK; ^14^ Department of Biological and Environmental Science University of Jyväskylä Jyväskylä Finland; ^15^ Department of Biology University of Virginia Charlottesville Virginia USA; ^16^ Department of Zoology Stockholm University Stockholm Sweden

**Keywords:** bulk RNAseq, cellular heterogeneity, deconvolution, gene expression, single‐cell RNAseq, transcriptomics

## Abstract

RNA sequencing (RNAseq) methodology has experienced a burst of technological developments in the last decade, which has opened up opportunities for studying the mechanisms of adaptation to environmental factors at both the organismal and cellular level. Selecting the most suitable experimental approach for specific research questions and model systems can, however, be a challenge and researchers in ecology and evolution are commonly faced with the choice of whether to study gene expression variation in whole bodies, specific tissues, and/or single cells. A wide range of sometimes polarised opinions exists over which approach is best. Here, we highlight the advantages and disadvantages of each of these approaches to provide a guide to help researchers make informed decisions and maximise the power of their study. Using illustrative examples of various ecological and evolutionary research questions, we guide the readers through the different RNAseq approaches and help them identify the most suitable design for their own projects.

## 1 INTRODUCTION

Understanding phenotypic diversity and adaptation is a key goal of evolutionary and ecological research. Most phenotypes have a complex genetic basis that depends on the effects of a large number of genetic loci (sometimes thousands; Barton, 2022; Boyle et al., 2017), which are expressed in interaction with the environment. Gene transcription represents the first step towards translating genotype information into phenotypes, and its products interact in complex gene regulatory networks and signalling cascades. Moreover, the transcriptome integrates a range of genetic, environmental and physiological signals (e.g. Buchberger et al., 2019; Everett et al., 2020; Hill et al., 2021). Accordingly, evolutionary change in spatial and temporal patterns of gene expression is one of the main drivers of phenotypic differentiation (e.g. Barbosa‐Morais et al., 2012; Brawand et al., 2011; Fukushima & Pollock, 2020; Gerhart & Kirschner, 2007; Mantica et al., 2024; Shapiro et al., 2004; Steiner et al., 2007). Gene expression analyses based on RNA sequencing (RNAseq) provide a powerful tool to study a wide range of ecological and evolutionary questions (reviewed e.g. in Oppenheim et al., 2015; Stark et al., 2019), in particular since they allow quantification of gene expression in organisms without high‐quality reference genomes (Chalifa‐Caspi, 2021; Cheng et al., 2018; Haas et al., 2013). Still, RNAseq analyses certainly profit from the availability of genome references. For instance, quantification of gene expression strongly depends on the quality of the available genome assembly and annotation (Torres‐Oliva et al., 2016).

Isolation of RNA is the starting point for any RNAseq experiment. For many organisms, such as vertebrates and plants, RNA is usually isolated from specific organs, body parts or tissue samples for RNAseq because the whole body is too large. For smaller organisms, such as small arthropods or other invertebrates, one can also choose to conduct gene expression analysis on RNA extracted from the whole body. Although possible and also done, tissue‐specific RNAseq is often more technically challenging for small organisms, and whole body RNAseq has therefore been common practice. In either case, one major complication of gene expression analyses in multicellular organisms lies in the large number of different cell types present within whole bodies, body parts and even specific tissues. For instance, the small nematode worm *Caenorhabditis elegans* is composed of 959 somatic cells, and the 302 cells of the nervous system fall into 128 different neuron types (Taylor et al., 2021). The human body consists of more than 3 × 10^13^ cells (Bianconi et al., 2013), and the first 400 cell types of 24 different tissues have only recently been characterised (Tabula Sapiens et al., 2022). Accordingly, RNAseq of whole bodies, body parts or tissues (i.e. bulk RNAseq) is based on a mixture of cells with specific expression patterns. This implies that bulk RNAseq analyses reflect the gene expression averaged across multiple cell types with distinct expression patterns. Recently developed single‐cell RNAseq (scRNAseq) approaches allow quantifying expression at the cellular level to elucidate differences among multiple cell types, thereby presenting an alternative to bulk RNAseq approaches (e.g. Alfieri et al., 2022; Nguyen et al., 2018; Wang, Sun, et al., 2021).

It is often difficult to decide whether it is advantageous to study gene expression in specific tissues, body parts, a collection of organs, entire bodies or individual cells. In this opinion article, we highlight major technical and methodological advantages and limitations of gene expression studies based on whole bodies, organs and tissues (bulk RNAseq), as well as of recent scRNAseq methods. We propose guidelines for typical research questions in ecology and evolution, and we highlight how scRNAseq can enhance the merit of future and existing bulk RNAseq datasets, in particular when combined with whole body RNAseq.

## 2 WHOLE BODY RNASEQ FOR A SYSTEMIC OVERVIEW OF GENE EXPRESSION

**FIGURE 1:**
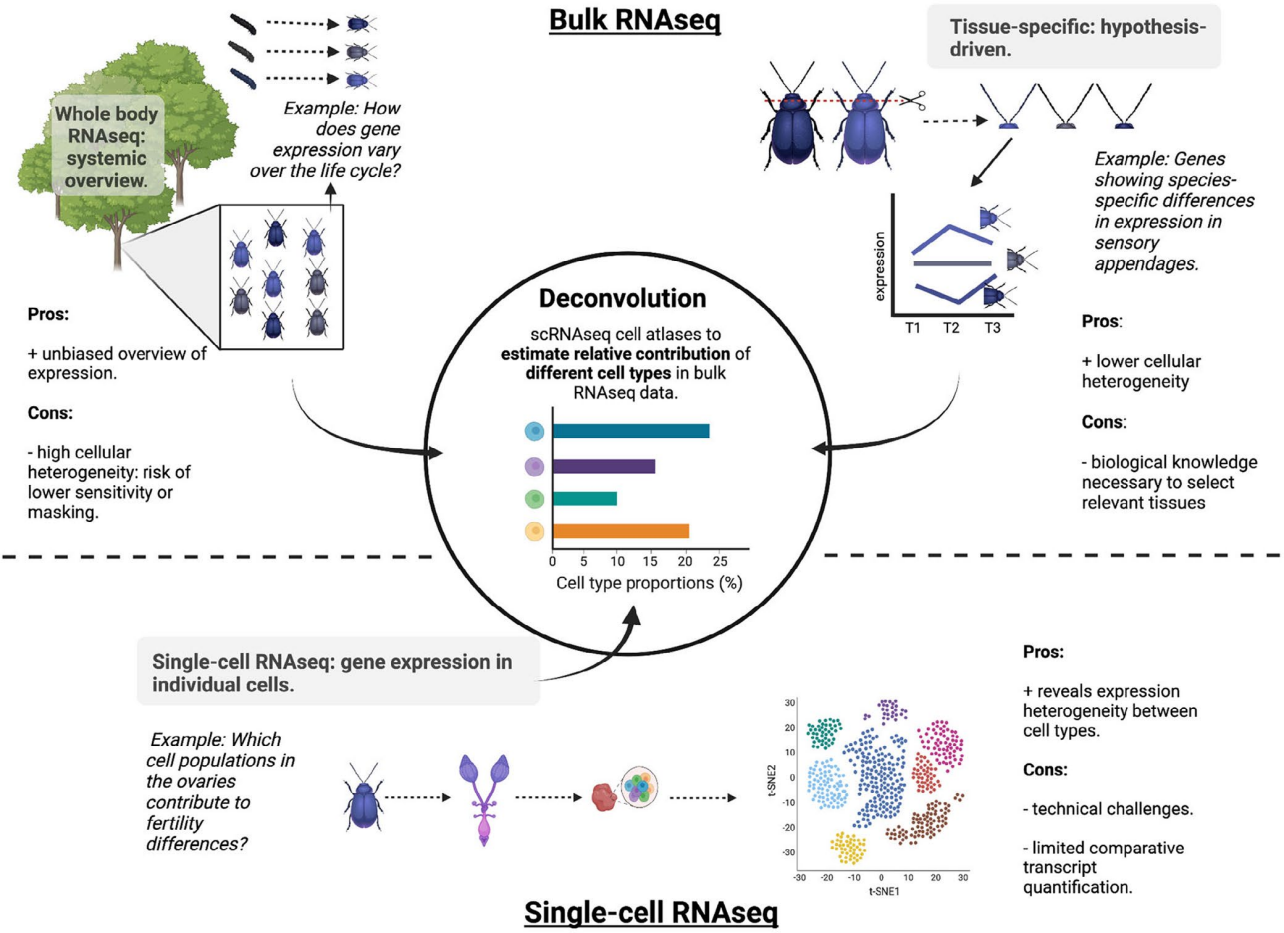
Advantages and disadvantages of RNAseq approaches, and opportunities for deconvolution. Each RNAseq approach, using either whole bodies, body parts or specific tissues (i.e. bulk RNAseq), or single cells (i.e. single‐cell RNAseq), has unique advantages and disadvantages that make them more or less suitable to address particular questions in the fields of ecology and evolution. Integrating different approaches can provide additional advantages, by harnessing the strengths of each approach. Here, we highlight deconvolution, by combining bulk and single‐cell RNAseq data, as a promising opportunity that has recently opened up for ecological and evolutionary research.

Bulk RNAseq methods depend on the principle that RNA is isolated from a heterogeneous set of cells, such as whole bodies or parts of it, and then sequenced in bulk. Whole body RNAseq has been especially popular in studies of small organisms (e.g. Bouvaine et al., 2012; Crawford et al., 2010; Teets et al., 2013; Winbush et al., 2012), as it is cost‐effective and it can be challenging to obtain sufficient high‐quality RNA from only a specific tissue of an individual. As whole body RNAseq provides an overview of the averaged gene expression patterns across all cells within an individual, the major advantage of this approach lies in the potential to provide a systemic overview of gene expression across the entire organism (Figure 1). Whole body RNAseq is therefore especially powerful when studying the evolutionary or ecological responses of phenotypes without a priori expectations about affected tissues, cell types, or specific physiological mechanisms. For example, studies of evolved and plastic responses underlying thermal adaptation have uncovered a diversity of physiological processes, which suggests broad systemic effects that depend on different tissues and cell types (e.g. Hsu et al., 2020; Kankare et al., 2016; Koniger & Grath, 2018; Mallard et al., 2020; Parker et al., 2021). In this case, focussing on specific tissues not only results in an incomplete analysis, but it could also potentially introduce a substantial bias if a non‐representative tissue is selected, affecting subsequent interpretation. Nevertheless, whole body RNAseq also comes with its specific challenges and considerations, which we briefly discuss below.

### 2.1 Genetic heterogeneity and pooling

Depending on the size of the organism (parts of) multiple individuals may be pooled prior to RNAseq library preparation. If all individuals have an identical genotype and are derived from the same environment, pooling clearly provides a benefit because stochastic inter‐individual variation is reduced. Pooling individuals with different genotypes is frequently done when genetically identical individuals are not available. If many genetically distinct individuals are pooled, a reliable and representative average gene expression level of the population can be obtained. The drawback of this approach is that information about the heterogeneity in gene expression in the population, or differences in heterogeneity between populations, is lost. Reliable estimates of variance require very large population samples, however, which are often not possible due to limited access to material and restrictions in research budget. We assume that pooling individuals for RNAseq approaches comes with challenges that also apply for Pool‐Seq of genomic DNA (e.g. Futschik & Schlötterer, 2010) and we propose that expression variation due to genetic heterogeneity warrants more attention in future research.

### 2.2 Sensitivity

All bulk RNAseq approaches measure average mRNA abundance among all cells in a whole body or specific tissue. Thus, changes affecting only a small number of cells might be difficult to detect against the background of the rest of the body (or tissue) with no expression changes (Kuhn et al., 2012). For example, a comparative analysis of expression patterns of honey bee sting glands and digestive tract versus whole abdomen demonstrated that 81% and 69% of significantly differentially expressed genes in these two tissues, respectively, were not detected as differentially expressed in the composite tissue (Johnson et al., 2013). Likewise, in a study on diabetes in humans, 35% of eQTLs identified from pancreatic islet data could not be detected when analysing whole pancreas data (Vinuela et al., 2020). In both cases, a plausible explanation is that some expression differences are limited to particular cell types, which become more difficult to detect when other cell types are present in the RNA extract.

One possible solution to improve sensitivity is to increase either coverage or, preferentially, the number of biological replicates. For example, Jaksic et al. (2020) measured whole body gene expression in *Drosophila simulans* that had been experimentally evolved under either warm or cool conditions. Using a relatively large sample size of 20 populations (10 warm‐evolved, 5 cool‐evolved and 5 ancestral) from which they sequenced a pooled RNA sample from the whole body of 50 males each, they identified differences in gene expression in dopaminergic neurons in the brain. Although these neurons constitute only a small percentage of the cells of the entire body, significant differences in gene expression could be identified and functionally validated using RNA interference mediated knockdown and pharmacological intervention (Jaksic et al., 2020). Hence, given sufficient power to detect small changes, bulk RNAseq of whole bodies can be a promising discovery tool even if only a subset of the cell population is affected.

### 2.3 Masking of signals in opposite directions

Many genes have pleiotropic functions, and are expressed in multiple cell types. For example, systematic studies of yeast found that more than 50% of tested mutations affected two or more organismal phenotypes (Dudley et al., 2005; Mattiazzi Usaj et al., 2020). Given widespread pleiotropy, in combination with transcriptional heterogeneity among cell types, it is conceivable that the experimental conditions studied might affect gene expression in multiple tissues or cell types, but in opposite directions. As bulk RNAseq only provides average expression levels of transcripts across all tissues and cells, such changes may partly or completely mask one another, essentially cancelling each other out so that no overall expression difference can be detected for a certain gene. Although this scenario is possible and warrants a careful interpretation of the outcomes of bulk RNAseq experiments, we expect that the exact cancellation of expression differences is unlikely in most cases. The previously discussed comparative study on honey bees indicated that a small proportion (<3%) of the differentially expressed genes exhibited an opposite differential expression pattern in the tissue‐specific samples versus the whole abdomen, which could theoretically obscure interpretation of the results if the expression patterns cancel each other out completely (Johnson et al., 2013). However, if differences in gene expression are only partially masked by cell type heterogeneity in bulk RNAseq experiments, it will often still be possible to detect these differences when a sufficiently powerful experimental design (number of replicates and sequencing depth) is used.

### 2.4 Allometric changes

Expression differences in whole body RNAseq samples could have two different causes, which are not mutually exclusive and could both represent adaptive responses: expression might differ within one or more cell types, or the relative proportion of different cell types in the whole body might be different. Relative size, in other words pleiotropy, allometry, of specific tissues (each differing in their gene expression profiles) can therefore be a key determinant of whole body RNAseq results, which is important to consider alongside transcriptional regulation. A frequently observed allometric change in evolutionary and ecological studies concerns, for example, the relative size of reproductive tissues. Here the allometric changes can be a mechanism by which an organism adapts to its environment; for example, males of polygamous species regularly increase testes size under increased sperm competition (Montgomery & Mank, 2016 and references therein). A point of concern is that allometric relationships could also lead to false negatives, when changes in expression occur but are offset by decreases in tissue size (Abell et al., 2022).

Hsu et al. (2020) proposed an approach to disentangle the effects of allometry versus transcriptional regulation on expression levels. They observed that ovaries of female flies that had evolved in a novel warm environment were enlarged, which made it difficult to draw conclusions on the evolution of transcriptional regulation. To overcome this limitation, Hsu et al. (2020) measured gene expression in gonads and carcasses of the same flies and determined a measure for allometric change, which was then used to correct the whole body expression data. This example shows that whole body RNAseq alone or in conjunction with tissue‐specific RNAseq can be a powerful, unbiased approach to identify evolutionary or ecologically relevant expression changes, which tissue‐specific RNAseq alone might fail to identify. Alternatively, tissue‐specific RNAseq in conjunction with quantitative estimates of the relative contribution of that tissue between conditions (e.g. populations, environments and treatments) could also distinguish between regulatory evolution and allometric differences (Buono et al., 2021). This could be achieved with fluorescent imaging (e.g. immunohistology) or transgenic cell labelling methods in combination with fluorescence‐activated cell sorting. In either case, it is important to recognise that both allometric and gene regulatory mechanisms can account for observed differences in whole body RNAseq data, but both mechanisms represent a genuine adaptive response that can be uncovered by whole body RNAseq.

## 3 FROM SYSTEMIC TO HYPOTHESIS‐DRIVEN RNASEQ

The evolution of complex body plans of multicellular organisms and their functions are intricately linked to tissue‐specific transcriptomic differentiation (Barbosa‐Morais et al., 2012; Brawand et al., 2011; Fukushima & Pollock, 2020; Mantica et al., 2024). For some types of research questions, a defined morphological structure or tissue may be of interest, in particular when specific target tissues responsible for the adaptive mechanism(s) have already been identified. In these cases, RNAseq of specific tissues and cell types may be the most logical step because the smaller number of different cell types increases the power to detect relevant gene expression differences (Figure 1). Also, complex phenotypic adaptations can depend on divergent transcriptional changes in different tissues, which might be missed when using a whole body RNAseq approach. For example, Salvador‐Martinez et al. (2018) analysed spatial gene expression patterns from different *D. melanogaster* embryonic anatomical structures, which indicated that genes expressed in the digestive system and ectoderm‐derived structures are under selective constraint, while genes expressed in the germ line showed high rates of adaptive substitution.

The key prerequisite for a successful tissue‐specific RNAseq analysis is sufficient biological knowledge to select the relevant tissues for the question of interest. An example comes from analyses of post‐mating gene expression changes in female fruit flies, triggered by the male ejaculate. Various studies have focussed on detecting differential expression in the female reproductive tract and the ovaries, which is extensive and can even be specific for distinct sub‐tissues (e.g. Kapelnikov et al., 2008; Veltsos et al., 2022). At the same time, receptors for sex peptide, one of the most important components of the ejaculate triggering female post‐mating effects, have been found throughout the female body, including in the abdominal ganglia and the brain. This indicates that key targets of sex peptide are located in tissues outside the female reproductive system (e.g. Gioti et al., 2012; Pasquier & Robichon, 2022; Yapici et al., 2008) and would be missed by a tissue‐specific RNAseq study. Hence these examples illustrate the power of a combined application of whole body and tissue‐specific RNAseq. As with all bulk RNAseq approaches, tissue‐specific RNAseq estimates gene expression of mixed cell types, and hence the technical limitations discussed above, also apply to tissue specific RNAseq, although to a lesser extent due to lower cellular heterogeneity within tissues as compared to whole bodies.

## 4 SINGLE‐CELL RNASEQ: CAPTURING GENE EXPRESSION DIFFERENCES AMONG CELLS

Recent single‐cell sequencing methods (scRNAseq) can address some of the limitations of bulk RNAseq by measuring gene expression in large numbers of individual cells (e.g. Alfieri et al., 2022; Nguyen et al., 2018; Wang, He, et al., 2021). By revealing the heterogeneity in expression between cells, as well as variation in transcriptional profiles among species, populations and experimental conditions, scRNAseq has great potential to uncover mechanisms underpinning ecologically or evolutionarily salient phenotypic variation (Figure 1). For instance, scRNAseq was recently applied to fat bodies in fruit flies to uncover the cellular mechanisms underlying the trade‐off between reproduction and immunity. It was demonstrated that limitations in the capacity of a specific subset of fat body cells to produce proteins constrains the immune response in reproducing females (Gupta et al., 2021). Despite its promises, scRNAseq comes with challenges that warrant careful consideration during the project planning phase.

### 4.1 Sample availability and preparation

A typical scRNAseq workflow requires access to at least 50,000 live cells (Pollen et al., 2014) and the availability of fresh material is often limited in ecological or evolutionary studies that deal with field samples. Potential solutions could be the sequencing of single nuclei (snRNAseq), which can be isolated from flash frozen tissue samples to allow some level of tissue conservation (Denisenko et al., 2020; Wiegleb et al., 2022). Similarly, simultaneous cell dissociation and fixation has been applied to preserve tissue samples for scRNAseq (Garcia‐Castro et al., 2021). However, obtaining sufficient material for small organisms or tiny tissue samples requires pooling of multiple individuals, which potentially introduces bias due to genetic heterogeneity (see Section 2.1 above). As the efficiency of capturing different cell types from a heterogeneous tissue varies considerably due to differences in cell size or cell shape, technical bias in cell type composition is expected (Darmanis et al., 2015; Yim et al., 2022). Thus, accurate and repeatable outcomes depend critically on the workflow used for sample preparation. Optimal parameters are highly tissue‐ and species‐specific, which may necessitate laborious and often costly empirical optimisation, especially for non‐model organisms (Svensson et al., 2018).

### 4.2 RNA content and gene coverage

Each individual cell comprises very little RNA per gene resulting in high sampling variation and thus uncertainties for transcript quantification. This limitation means that, if no expression of a gene is detected in a certain cell, it is almost impossible to distinguish between a biological explanation (i.e. a gene is indeed not expressed) and a technical one (i.e. no reads due to inefficient sampling). The occurrence of such null data (also called dropout events) is much higher in scRNAseq compared to bulk RNAseq data (Bacher & Kendziorski, 2016). scRNAseq methods differ significantly in the number of genes that can unequivocally be detected per cell and the number of cells that can be analysed (Ziegenhain et al., 2017). For instance, the Smart‐Seq2 method can detect many genes including low abundance transcripts in a few hundred cells in one run, while the 10× genomics method can process up to 10,000 cells per run, but with a higher noise level for low abundance transcripts (Wang, He, et al., 2021). While novel methods based on multiple rounds of in‐cell barcoding (i.e. combinatorial indexing; e.g. split‐pool ligation‐based transcriptome sequencing (SPLiT‐seq)) (Cao et al., 2019; Conte et al., 2023; Rosenberg et al., 2018) may mitigate this trade‐off in the near future, scRNAseq applications are still limited for comparative transcript quantification. This is especially important to consider if the expected expression differences are rather small (Zhang et al., 2020), which is often the case in ecological or evolutionary research, for example, for studies examining environmental effects on individuals of the same species.

### 4.3 Need for well‐annotated reference genomes

The commonly used scRNAseq methods rely on sequencing the 3‐prime ends of captured fragments (Ziegenhain et al., 2017), which can only be linked to particular genes if a well‐annotated reference genome is available. Moreover, in snRNAseq nascent RNA is sequenced as well as mature mRNA, resulting in about one quarter of all reads originating from introns (Grindberg et al., 2013). Those reads can only be unequivocally assigned to a gene if the reads are mapped against a well‐annotated genome, which is often unavailable for non‐model systems used in ecological or evolutionary research. This is in contrast to bulk RNAseq data, which covers entire transcripts facilitating the de novo assembly of reference transcriptomes for subsequent transcript quantification (Grabherr et al., 2011; Haas et al., 2013; Raghavan et al., 2022), avoiding the need for a reference genome. Hence, differential gene expression analyses based on bulk RNAseq are readily applicable for non‐model organisms without the need for a reference genome.

In light of the outlined technical limitations and significantly higher costs, we think that scRNAseq has not yet reached the required maturity for a routine application in most research in ecology and evolution. In the following section, however, we discuss how single cell expression atlases obtained by shallow sequencing of a large number of cells can be used to enhance the interpretation of existing and future whole body or tissue‐specific RNAseq data.

## 5 DECONVOLUTION: MERGING TWO WORLDS

Biological interpretation of gene expression data from a heterogeneous cell population is the biggest challenge for bulk RNAseq. To overcome this problem, various approaches to assess whether differentially expressed genes are enriched for certain molecular or biological functions (i.e. gene ontology (GO) enrichment) (Gene Ontology et al., 2023; Thomas et al., 2022) or for specific signalling pathways (i.e. pathway enrichment) (Reimand et al., 2019) are routinely applied. Such functional enrichment analyses can help to extract biologically meaningful hypotheses or pinpoint candidate cell types or tissues from the often large numbers of differentially expressed genes (e.g. Stanford et al., 2020). These analyses can readily be applied in many model organisms for which databases with gene‐to‐function information are available. For instance, a study on the evolution of gene expression variance in flies used this approach to show that a small set of genes with a significant loss in expression variance was over‐represented among genes with catabolic function and expression in the gut, suggesting that gene expression evolved in response to a less variable diet in the laboratory (Lai & Schlotterer, 2022). Similarly, Green et al. (2022) identified differentially expressed genes in response to copper exposure using whole body RNAseq of natural *Drosophila melanogaster* populations. A GO enrichment analysis identified the midgut as a candidate tissue and established a link between the preservation of gut acidity and tolerance to copper (Green et al., 2022). However, to leverage such functional databases for non‐model organisms, a solid gene orthology assignment must be established prior to the enrichment analyses. While functional enrichment analyses are often the only way to extract biological information from gene lists, they depend on current knowledge, which is inherently incomplete and biased (Dessimoz & Skunca, 2017).

scRNAseq data provides exciting new opportunities to extract biological information from lists of differentially expressed genes obtained from bulk RNAseq approaches. The assessment of transcriptional profiles of distinct cell types has resulted in the development of single cell atlases across tissues, conditions, developmental stages (e.g. Allen et al., 2020; Brunet Avalos et al., 2019; Chen et al., 2021; Corrales et al., 2022; Hu, Comjean, et al., 2021; Hu, Tattikota, et al., 2021; Karaiskos et al., 2017; Li et al., 2022; Najle et al., 2023; Papatheodorou et al., 2020; Suo et al., 2022; Wang, Sun, et al., 2021; Xu et al., 2021) and even for entire organisms (Li et al., 2022). Such single cell atlases can directly be used to ask whether genes identified in bulk RNAseq approaches may be expressed in specific cell types. Moreover, deconvolution methods have been established, which estimate the relative contribution of different cell types in bulk RNAseq datasets (Mohammadi et al., 2017; Venet et al., 2001). When information about the gene expression profile of each cell constituting a bulk RNAseq sample is available, it is possible to decompose the average gene expression value into the expression of individual cell types that share an expression profile (Figure 1). Most expression deconvolution methods require prior information about cell‐type specific marker genes (Zaitsev et al., 2019) or expression profiles encompassing multiple genes (Avila Cobos et al., 2018, 2020), whose expression level is directly correlated with the abundance of that cell type in a heterogeneous tissue. While such cell‐type specific marker genes or expression profiles are typically identified based on extensive prior knowledge in model organisms (e.g. spatial expression data and functional assays), scRNAseq now allows the identification of such markers for many non‐model systems too (e.g. Andrade Barbosa et al., 2021).

Expression deconvolution has successfully been applied to estimate the dynamics of cell type composition in evolving yeast populations exposed to environmental stress, the induction of extensive DNA damage and during sexual reproduction (i.e. sporulation) (Lu et al., 2003). In another eco‐evolutionary study, a scRNAseq analysis of three populations of three‐spined stickleback fish (*Gasterosteus aculeatus*) that exhibit natural variation in parasite resistance revealed differences in the composition and cell‐type specific expression profiles of immune cells. Moreover, immune cell‐type specific marker genes identified using the scRNAseq data were used to re‐analyse previous tissue‐specific RNAseq datasets of F_2_ crosses to show that the response of gene expression in antigen‐presenting cells to infection is most likely the result of regulatory variation and not due to an increase in the number of antigen‐presenting cells (Fuess & Bolnick, 2023). Combining single cell expression atlases with bulk RNAseq datasets to estimate the expression profiles of individual cell types thus provides an important extra layer of biological insight that can be obtained from whole body or tissue‐specific RNAseq datasets. We argue that the impact of deconvolution methods is largest for complex tissues or even whole bodies, and can mitigate some of the drawbacks associated with bulk RNAseq approaches, as discussed above. As such, the combination of whole body RNAseq with scRNAseq has the potential to bring together the best of both worlds by combining a systemic overview of gene expression with cell‐type specific information.

## 6 CONCLUSIONS AND RECOMMENDATIONS

In light of such exciting opportunities, but also the challenges of different RNAseq approaches, it is often not easy to decide which strategy is best suited for a certain research question. The first important consideration is the phenotype that is being studied. For instance, if one wants to identify genes underlying the evolution of the morphology of a specific organ, such as the formation of pigment spots on insect wings (Hanly et al., 2019; Wee et al., 2023) or the size and shape of beetle horns (Emlen et al., 2007; Ohde et al., 2018) it is logical to restrict RNAseq analyses to that specific tissue. Similarly, the evolution of many behavioural traits is for an important part likely linked to variation in the composition and function of the central nervous system (CNS). Therefore, CNS‐specific RNAseq experiments will be powerful to identify meaningful candidate genes (even though the CNS itself is a complex tissue with multiple cell types performing distinct roles). We caution, however, that often phenotypes cannot be restricted to single tissues. A nice example for this challenge comes from phenotypes related to the stress response (Horvath et al., 2023) or life history (Rodrigues et al., 2021) of an organism, where more systemic transcriptomic changes involving multiple organs and tissues are expected. In such cases, whole body RNAseq analyses could limit the bias due to missing biologically important signals.

A second important consideration is the level of prior knowledge, available tools and access to annotated genomes of the organism of study. While extensive mechanistic details are publicly available for many organs in model organisms, such data are often missing for non‐model systems. If no hypotheses about specific tissues or cell types underlying a certain phenotypic trait are available, a whole body bulk RNAseq approach provides a systemic and comprehensive overview of potential gene expression changes. If putative target tissues have been identified based on prior whole body expression data or based on similar phenotypes in other models, tissue‐specific RNAseq has the potential to assess variable expression of genes with minute differences in expression or lower overall expression levels, which may be missed in whole body RNAseq.

A third consideration is the opportunity to combine different RNAseq approaches to strengthen the biological interpretation of RNAseq data, while diminishing the inherent weaknesses of each individual approach. The combination of whole body RNAseq to establish first hypotheses followed by integration of tissue‐specific expression information has been successfully applied in ecological and evolutionary research to identify mechanisms of phenotypic variation (Abbott et al., 2020; Lai & Schlotterer, 2022). Moreover, with increasing availability of single‐cell expression data for model organisms and non‐model organisms alike, existing and future bulk RNAseq data can now be (re‐)interpreted to gain novel insights into tissue‐ and cell‐type specific gene expression divergence. Most importantly, expression deconvolution methods have a great potential to distinguish between regulatory evolution and allometric differences, respectively, observed in bulk RNAseq data. We argue that future eco‐evolution research would profit from a community driven development of tissue databases and single cell atlases for entire small organisms or specific tissues to facilitate the identification of cell‐type specific or condition‐dependent markers. Such reference databases and markers will allow a better interpretation and integration of future bulk RNAseq data, which is still the best accessible and often most informative technology for most questions in eco‐evolution research.

In conclusion, we argue that both bulk RNAseq, whether whole body, body‐part or tissue‐specific, and scRNAseq have unique advantages and disadvantages. The best choice of RNAseq approach depends strongly on the model system, prior knowledge of the phenotype, and the biological level of interest (e.g. gene regulatory networks, physiology, allometry or phylogenies). Integrating different RNAseq methods allows to harness the strengths, versatility and opportunities of each approach to study research questions in ecology and evolution.

## AUTHOR CONTRIBUTIONS

Conceptualisation: KMH, SG, NP, MGR, CS, EDM, JKA, TF, AG‐S, JG, LH, MK, BL, VMY, LY; Visualisation: LY; Writing – Original Draft Preparation: KMH, SG, NP, MGR, CS; Writing – Review and Editing: KMH, SG, NP, MGR, CS, EDM, JKA, IA, MC‐Z, TF, CF, AG‐S, JG, LH, MK, BL, DJO, RRS, VMY, LY.

## CONFLICT OF INTEREST STATEMENT

The authors declare that there is no conflict of interest.

## OPEN RESEARCH BADGES

This article has earned an Open Data badge for making publicly available the digitally‐shareable data necessary to reproduce the reported results.

## Data Availability

Not applicable.
